# Appendiceal mucinous neoplasm with myxoglobulosis occurring 3 years after perforated barium appendicitis: a case report

**DOI:** 10.1186/s40792-019-0665-y

**Published:** 2019-07-02

**Authors:** Takatsugu Fujii, Shigeo Toda, Naoko Inoshita, Kenji Tomizawa, Yutaka Hanaoka, Shuichiro Matoba, Hiroya Kuroyanagi

**Affiliations:** 1 Department of gastrointestinal surgery, Toranomon Hospital, 2-2-2 Toranomon, Minato-ku, Tokyo, 105-8470 Japan; 2 Department of pathology, Tokyo Metropolitan Geriatric Medical Center, 35-2 Sakaecho, Itabashi-ku, Tokyo, 173-0015 Japan

**Keywords:** Appendiceal mucinous neoplasm, Myxoglobulosis, Barium appendicitis, Laparoscopic ileocecal resection

## Abstract

**Background:**

Myxoglobulosis is considered a subtype of appendiceal mucinous neoplasm (AMN). Factors affecting the occurrence of myxoglobulosis include proximal appendiceal obstruction and mucosal secretion at the residual appendiceal mucosa. In addition, myxoglobulosis has also been reportedly associated with persistent chronic inflammation. We report a case of AMN with myxoglobulosis occurring 3 years after perforated barium appendicitis and the importance of caution during surgery for barium peritonitis and elucidate the pathology of myxoglobulosis.

**Case presentation:**

A 45-year-old man with an AMN underwent laparoscopic ileocecal resection 3 years after peritonitis caused by perforated barium appendicitis. The patient had a medical history of perforated barium appendicitis after barium swallow imaging, which was treated conservatively 3 years ago. Computed tomography (CT) revealed cystic enlargement of the appendix and remnant barium around the appendix. He was then pathologically diagnosed with a low-grade AMN based on the resected specimen, and the appendix filled with white globules was diagnosed as myxoglobulosis. When barium is not absorbed, it causes chronic inflammation. As barium was observed around the appendix, prolonged inflammation, and appendicitis may have contributed to the myxoglobulosis. The circumference of the appendix firmly adhered to the surrounding tissue with barium; hence, it was difficult to perform appendectomy. Barium that enters the anastomotic site causes stenosis of this part; therefore, excision of the ileocecal region in the intestinal part where barium is not present was selected instead of appendectomy. Colonoscopy performed 1 year after surgery and showed no evidence of anastomotic stricture.

**Conclusion:**

This case suggested that barium peritonitis caused strong adhesions with the surrounding tissue; thus, careful manipulation was necessary to avoid perforating the appendix. Appendectomy and partial cecal resection were predicted to be difficult because of adhesion by barium. In addition, the ileocecal resection was selected because we had to choose an anastomotic site without barium. The perforated appendicitis caused stenosis of the appendix orifice, and barium surrounding the appendix caused persistent chronic inflammation. This was suggested to contribute to the myxoglobulosis.

## Background

Barium swallow test is known to be a risk factor for appendicitis [1], and appendix perforation may cause barium peritonitis [2–6]. The reported incidence of barium appendicitis is 1.19 per 1000 person-years [1]. On the contrary, myxoglobulosis is considered a subtype of appendiceal mucinous neoplasm (AMN) [7–9], with numerous white globules, which look like frog eggs, in the appendix [10]. The incidence of myxoglobulosis constituted 0.35–0.8% of appendiceal mucocele [10]. However, appendiceal myxoglobulosis associated with perforated barium appendicitis has not been previously reported.

Barium leaks into the peritoneal cavity, causing strong adhesions [11]. Furthermore, stenosis of the anastomosed site occurs when barium is involved in the anastomosis [12, 13].

In this report, we present a patient with AMN who underwent laparoscopic ileocecal resection 3 years after peritonitis caused by perforated barium appendicitis. We also emphasize the importance of caution during surgery for barium peritonitis and elucidate the pathology of myxoglobulosis.

## Case presentation

**Fig. 1:**
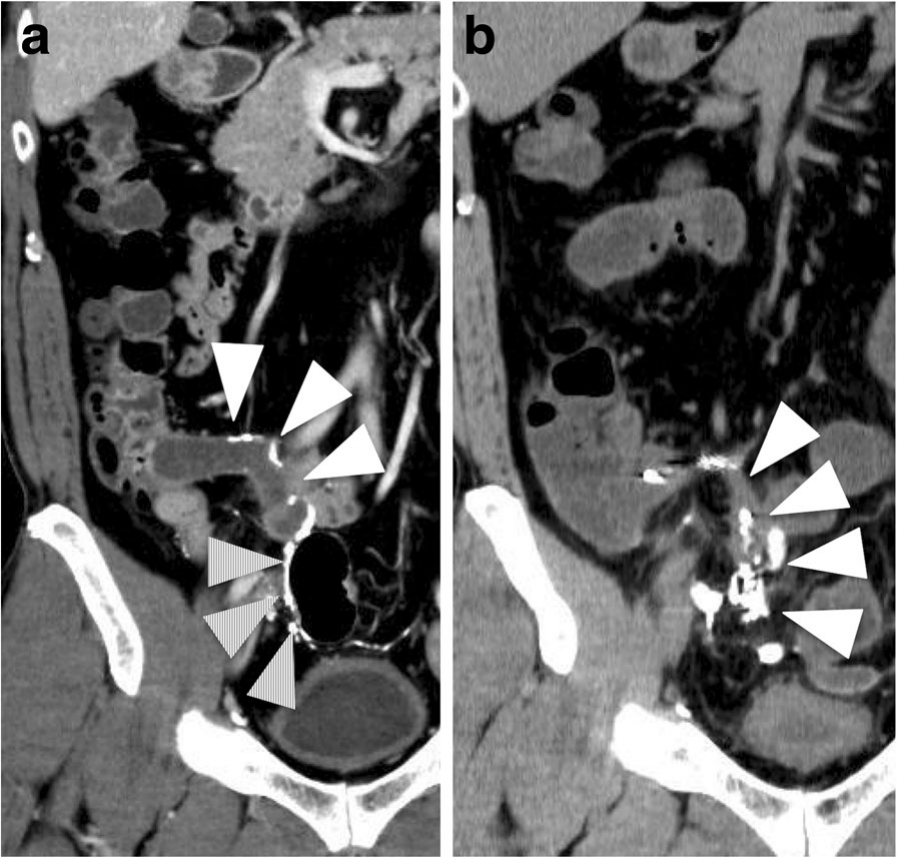
**a** Abdominal CT revealed a cystic lesion, measuring 10 × 3 cm, in the appendix and barium around the appendix (white triangle), and sigmoid colon (triangle with diagonal lines). **b** At the time of perforation of the appendix about 3 years ago, there was no finding of AMN, and barium leaked from the tip of the appendix

A 45-year-old man was diagnosed with AMN using colonoscopy and computed tomography (CT). He had a history of perforated barium appendicitis 3 years ago. Physical examination revealed no specific abdominal findings. The results of routine blood examination and serum tumor markers (carcinoembryonic antigen and carbohydrate antigen 19–9) were within normal limits. Colonoscopy revealed appendiceal intussusception to the cecum, caused by the mucocele of the appendix. Abdominal CT revealed a cystic lesion, measuring 10 × 3 cm, in the appendix and barium around the cecum, appendix, and sigmoid colon (Fig. 1a). No regional lymph node enlargement or metastasis was observed. At the time of perforation of the appendix 3 years ago, there was no finding of AMN, and barium leaked from the tip of the appendix (Fig. 1b).

We performed ureteral stent insertion, laparoscopic ileocecal resection. The ureteral stent made it easier to identify the ureter. Laparoscopic exploration revealed severe adhesions between the greater omentum and small intestines, appendix, and sigmoid colon including some barium cast. Preoperative CT revealed that barium remained around the ileocecal region. There was no barium nodule in the anastomotic region, and careful anastomosis was performed extracorporeally.

**Fig. 2:**
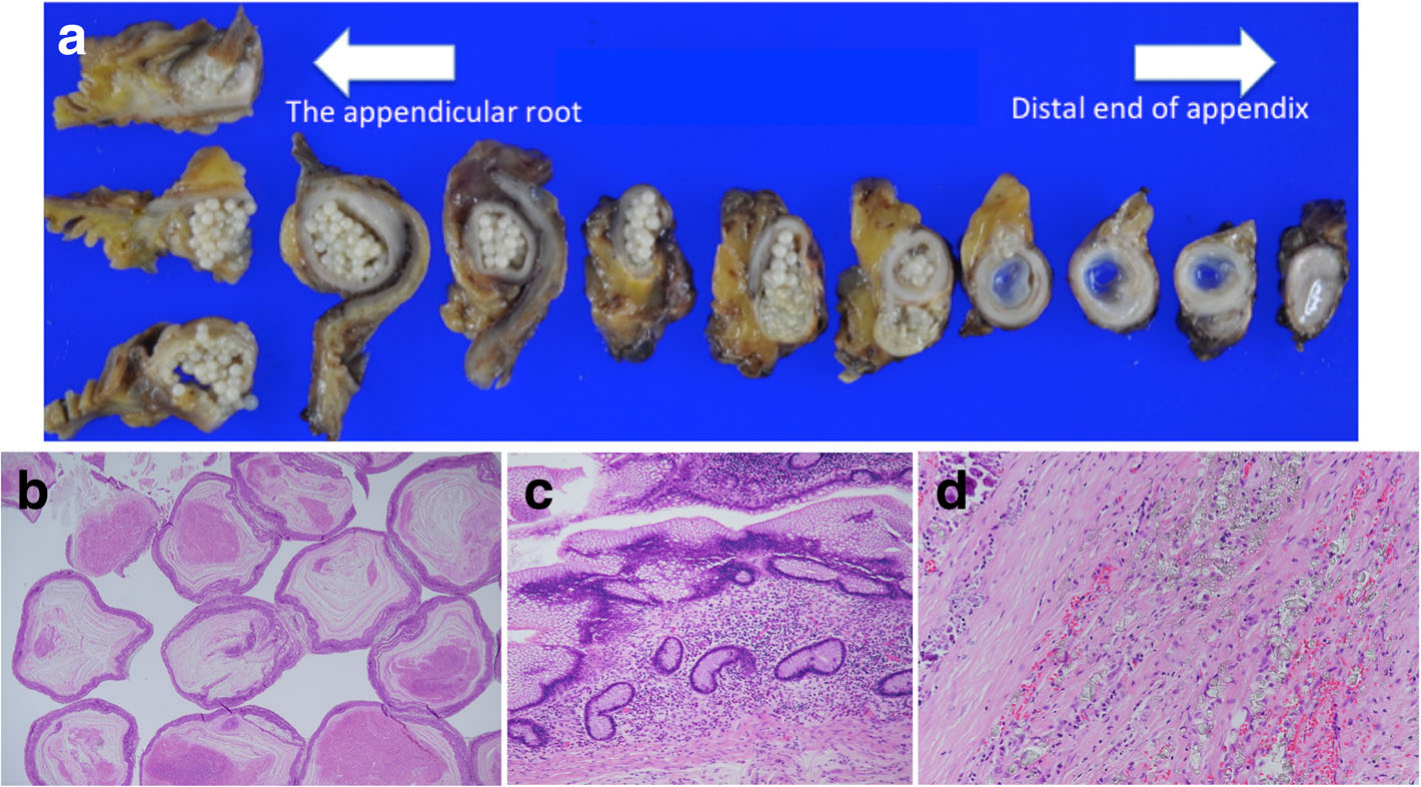
**a** The appendiceal lumen was full of frog egg-like 1–4 mm white globules. **b** The white globules consisted of thin laminations of mucin surrounding a granulated tissue. **c** The appendiceal lumen had low-grade adenoma-produced mucus. **d** Barium crystal was present in the granulated tissue in the appendiceal serosa

The total operative time was 363 min, and the blood loss was 50 mL. The resected specimen was pathologically diagnosed as a low-grade AMN with myxoglobulosis. The appendiceal lumen was full of frog egg-like 1–4 mm white globules (Fig. 2a). The white globules consisted of thin laminations of mucin surrounding a granulation tissue (Fig. 2b). The appendiceal lumen had a normal appendiceal epithelium and low-grade adenoma-produced mucus (Fig. 2c). The edematous change of the appendiceal tip and occlusion of the orifice of the appendix were thought to be caused by the perforated barium appendicitis. The appendix was surrounded by granulated and fibrous tissue with barium on the side of the appendiceal serosa (Fig. 2d), but no barium was found in the appendiceal lumen or white globules. Colonoscopy performed 1 year after surgery and showed no evidence of anastomotic stricture.

## Discussion

Barium appendicitis is a relatively rare disease. Barium accumulation in the appendix after barium swallow can cause appendicitis. If barium entrapped the anastomosis, it forms granulation and causes stenosis of the anastomotic site [13, 14]. In addition, barium leaks into the peritoneal cavity, causing prolonged peritonitis and inflammation [15, 16]. Barium peritonitis is said to develop in 2–8 cases per 10,000 barium swallow examinations [12].

Myxoglobulosis is a subtype of AMN, in which the appendiceal lumen is full of white globules that look like frog eggs. Factors affecting the occurrence of myxoglobulosis include proximal appendiceal obstruction and mucosal secretion at the residual appendiceal mucosa; in addition, some studies have reported myxoglobulosis to be associated with persistent chronic inflammation [9–11].

As perforation of an AMN can cause pseudomyxoma peritonei, it is important to avoid perforation of the appendix during surgery. Appendiceal resection or partial cecal resection is sufficient in case of an appendiceal mucinous cystadenoma [17]. The circumference of the appendix firmly adhered to the surrounding tissue with barium; hence, it was difficult to perform appendectomy. The ileocecal resection was selected because the anastomotic site had no barium. In particular, because the appendiceal tip was perforated once and the peripheral appendix and sigmoid colon adhered with barium, dissection required caution. The appendix adherent to the epiploic appendages of the sigmoid colon was carefully removed and resected en bloc*.*

Gastrointestinal surgery after barium peritonitis requires caution of anastomotic stenosis. According to Kitajima and Nishina, inflammatory granuloma was formed by entrapped barium in the anastomotic site, causing postoperative stenosis, which required anastomotic resection [13, 14]. At the anastomotic site, anastomosis at the part without barium and sufficient washing are important [13].

The globules consisted of faint eosinophilic laminations of mucin surrounding a necrotic tissue core, which is consistent with a previous report [11]; no barium was found in the appendiceal lumen or white globules. The barium remaining around the appendix possibly have caused chronic inflammation and contributed to the myxoglobulosis [15, 16] and the appendicitis caused root stenosis.

## Conclusion

The perforated appendicitis caused stenosis of the appendix orifice, and barium surrounding the appendix caused persistent chronic inflammation. This was suggested to contribute to the myxoglobulosis. Because of adhesion by barium, appendectomy and partial cecal resection were predicted to be difficult. In addition, ileocecal resection without the use of barium was done in the anastomotic site.

## Data Availability

The authors declare that all the data in this article are available within the article.

## Abbreviations

AMN: Appendiceal mucinous neoplasm
CT: Computed tomography
